# Use of fexinidazole in *gambiense* human African trypanosomiasis: a retrospective analysis of cases treated in Lui Hospital, South Sudan (2018–2024)

**DOI:** 10.1007/s15010-025-02633-6

**Published:** 2025-09-04

**Authors:** Francesca Mariotti, Riccardo Paggi, Matteo Basilico, Sofia Pettenuzzo, Grant Sebit Benson, Abiodun Amodu, Lorenzo Zammarchi, Stefano Rusconi, Chiara Scanagatta, Giovanni Putoto, Stefano Dacquino, Elena Gelormino

**Affiliations:** 1 Department of Experimental and Clinical Medicine, University of Florence, Florence, Italy; 2 Doctors with Africa CUAMM, Padua, Italy; 3 School of Public Health, Vita-Salute San Raffaele University, Milan, Italy; 4 Clinic of Infectious Diseases, Department of Health Science, ASST Santi Paolo e Carlo, University of Milan, Milan, Italy; 5 Lui Hospital, Mundri East County, Western Equatoria State South Sudan; 6 Doctors with Africa CUAMM, Juba, South Sudan; 7 Infectious Diseases Unit, Ospedale Civile di Legnano ASST Ovest Milanese, University of Milan, Legnano, Italy

**Keywords:** Human African trypanosomiasis, *Trypanosoma brucei gambiense*, Fexinidazole, Pentamidine/NECT, South Sudan

## Abstract

**Purpose:**

Fexinidazole, an oral molecule, replaced pentamidine and combined treatment with nifurtimox and eflornithine (NECT) therapy for stage 1 and non-severe stage 2 *gambiense* human African Trypanosomiasis (*g*-HAT), respectively. The study aims to evidence differences of outcome at discharge and adverse drug reactions (ADRs) between fexinidazole and pentamidine/NECT regimens in patients with stage 1 and non-severe stage 2 *g*-HAT admitted to Lui Hospital (Western Equatoria, South Sudan), a historical *g*-HAT focus.

**Methods:**

Data of patients (*n* = 86) admitted to Lui Hospital from July 2018 to June 2024 with *g-*HAT diagnosis were included. Among them, we considered for the analysis patients eligible for both fexinidazole and pentamidine/NECT regimens (i.e. patients without symptoms/signs compatible with severe stage 2 *g*-HAT).

**Results:**

In the study population 17% of patients were registered with an unfavourable outcome (signs or symptoms of *g*-HAT at discharge or death attributable to *g*-HAT or *g*-HAT treatment occurred during hospitalization). No significant differences between fexinidazole and pentamidine/NECT in terms of outcome at discharge (23% vs. 6%, *p* = 0.230) and ADRs frequency (70% vs. 50%, *p* = 0.181) were reported. Although fexinidazole cohort experienced more gastro-intestinal ADRs than pentamidine/NECT cohort (63% vs. 19%, *p* = 0.005), discontinuation of oral treatment has not been recorded.

**Conclusion:**

Patients treated with fexinidazole and pentamidine/NECT showed similar results in terms of outcome at discharge and ADRs, in line with current data available in literature. However, few real-life studies on efficacy of fexinidazole treatment were published: to our knowledge, this is the first one conducted in South Sudan.

**Supplementary Information:**

The online version contains supplementary material available at 10.1007/s15010-025-02633-6.

## Introduction

Human African Trypanosomiasis (HAT), or sleeping sickness, is a neglected tropical disease caused by parasites belonging to the genus *Trypanosoma brucei*; it is transmitted by tsetse fly (*Glossina ssp.*) and widespread in Sub-Saharan African countries [1, 2, 3].

The disease is characterized by two stages: haemo-lymphatic (stage 1) and meningo-encephalitic (stage 2) with central nervous system (CNS) involvement and neurological disturbances, including sleeping disorders (hence the name “sleeping sickness”) and with possible progression to coma and, ultimately, death. The infection is supposed to be fatal unless treated, and can be distinguished in a slowly progressive anthroponotic form caused by *Trypanosoma brucei gambiense* (*gambiense* HAT, *g*-HAT) in Western and Central Africa, and a faster progressive zoonotic form caused by *Trypanosoma brucei rhodesiense* (*rhodesiense* HAT, *r*-HAT) in Eastern and Southern Africa [1, 3, 4].

A strategy based on case-finding and curative treatment that interrupts transmission by depleting the reservoir of parasites in humans, combined with vector control activities [1, 2, 3, 5, 6], led to a gradual reduction in cases in recent years, with 799 *g*-HAT cases recorded worldwide in 2022 (corresponding to 97% reduction since 2000) [7]. As of February 2025, eight countries have been validated by WHO for eliminating *g*-HAT as a public health problem (Côte d’Ivoire, Togo, Benin, Equatorial Guinea, Uganda, Ghana [7], Chad [8], and Guinea [9]), making the interruption of transmission by 2030 a feasible target [1, 3, 6, 7, 10, 11]. However, *g*-HAT persists in many Sub-Saharan countries, such as South Sudan [12].

Diagnosis should be made as early as possible to avoid complications and follows a three-steps approach: screening, diagnostic confirmation, and staging. Serological screening consists of immunochromatographic rapid diagnostic test (RDT) or in the Card Agglutination Test for Trypanosomiasis (CATT). Diagnostic confirmation relies on finding trypanosomes in blood (using capillary tube centrifugation -CTC- or the Woo test) or lymph nodes. Staging consists of defining CNS involvement (stage 1 vs. stage 2) through cerebrospinal fluid (CSF) examination. In case of stage 2, the disease severity is determined based on CSF findings: non-severe stage 2 when CSF contains < 100 white blood cells (WBC)/ 𝜇L with or without evidence of trypanosomes, severe stage 2 when CSF contains > 100 WBC/𝜇L with or without evidence of trypanosomes [13, 14].

Historically, treatment for *g*-HAT relied on pentamidine (stage 1), nifurtimox and eflornithine combination therapy (NECT) and melarsoprol (stage 2 and relapses), characterized by partial or total parenteral administration. In 2019, WHO included fexinidazole as the first-line drug for stage 1 and non-severe stage 2; Food and Drug Administration approved the drug in 2021 for the same indication. The regimen is completely oral and reduces the need for systematic lumbar puncture (LP) for staging, which is necessary only when severe stage 2 is suspected. The evidences supporting the efficacy of fexinidazole were provided by a two-branch randomized clinical trial comparing fexinidazole with NECT [15] and by two open-label single-arm studies [16, 17]: the indication for stage 1 and non-severe stage 2 *g*-HAT was recently confirmed in 2024 WHO HAT guidelines [3].

Since the official introduction of fexinidazole in WHO guidelines, few studies have reported the real-life efficacy of the drug in *g*-HAT treatment [5, 17]. In December 2023 the European Medicines Agency adopted a positive opinion on fexinidazole for the treatment of *T. b. rhodesiense*, and recently the drug has been recommended as first-line therapy in both stage 1 and stage 2 *r*-HAT [3, 18]. In contrast, studies of fexinidazole for American trypanosomiasis did not report sufficient efficacy unless administered with a poorly tolerated posology [19, 20]. Moreover, fexinidazole seems to be useful in treating infections such as leishmaniasis, giardiasis and amebiasis [21].

To date, all the drugs used for *g*-HAT are freely donated and distributed by WHO to ensure that all *g*-HAT patients worldwide have access to the most effective treatment available [1, 3].

In South Sudan *g*-HAT cases are present in historically defined foci within a geographical belt between Kajo-Keji and Tambura, located in Equatoria area, where the vector *Glossina fuscipes fuscipes* is present [2, 22]. In particular, *g*-HAT prevalence in seven villages of Mundri East and Mundri West Counties in 2008 was 0.46% [23]. To date, no studies on the efficacy of fexinidazole in South Sudan are available.

## Materials and methods

### Study design and study population

This is a single-center, observational, and retrospective cohort study. All patients admitted to Lui Hospital with a diagnosis of *g*-HAT were included in the analysis, which was conducted from 01/07/2018 to 30/06/2024.

**Fig. 1:**
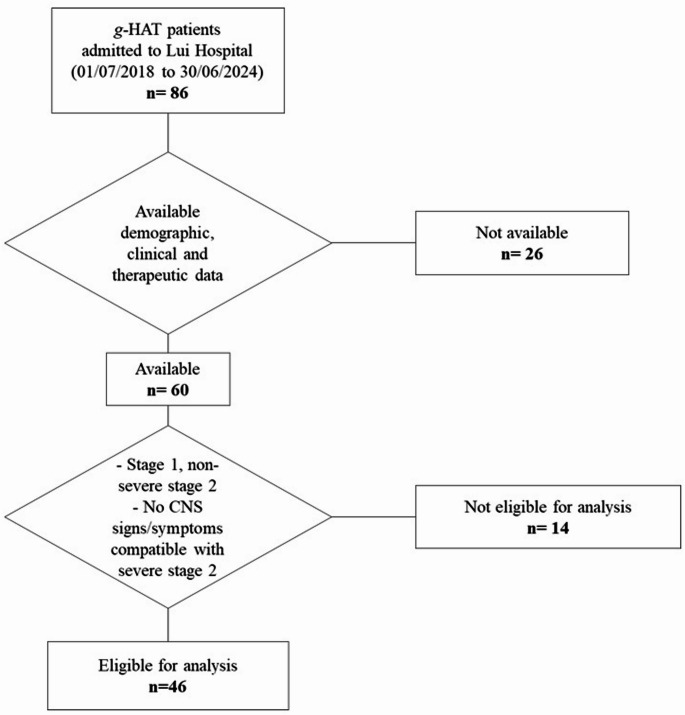
Flow diagram showing *gambiense* human African trypanosomiasis cases confirmed in Lui Hospital, Western Equatoria, South Sudan (July 2018-June 2024), and selection of patients according to availability of clinical/therapeutic data and eligibility for analysis Footnotes: CNS: central nervous system; *g*-HAT: *gambiense* human African trypanosomiasis

Demographic, clinical (neurological examination and symptoms assessment performed by medical doctors or clinical officers at admission and discharge) and therapeutic (treatment, adverse drug reactions -ADRs) characteristics were recorded for all patients, when available. Among 86 patients admitted in the hospital during the study period, complete demographic and clinical documentation was available for 60 patients. Patients eligible for fexinidazole (LP-defined stage 1 and non-severe stage 2 *g*-HAT, or patients without neurological signs or symptoms compatible with severe stage 2 *g*-HAT who did not undergo LP) were 46 (Fig. 1).

Available data were already present in Lui Hospital patients wards registers, pharmacy records, and HAT surveillance registers.

Strengthening the reporting of observational studies in epidemiology (STROBE) guidelines were used to perform the analysis and elaborate the manuscript (Supplementary Material).

### Aims of the study

1. The first aim of the study is to compare patients admitted to Lui Hospital with stage 1 and non-severe stage 2 *g*-HAT, treated with fexinidazole *vs* patients with the same stage of disease treated with pentamidine (stage 1) and NECT (non-severe stage 2), analyzing the outcome at discharge, defined as favourable (no signs and symptoms of *g*-HAT) and unfavourable (signs or symptoms of *g*-HAT or death related to *g*-HAT or *g*-HAT treatment occurred during hospitalisation).
2. The second aim of the study is to compare patients admitted to Lui Hospital with stage 1 and non-severe stage 2 *g*-HAT treated with fexinidazole *vs* patients with the same stage of disease treated with pentamidine (stage 1) and NECT (non-severe stage 2), analyzing the frequency of ADRs during treatment.

For both study aims, the analysis was conducted only on patients with stage 1 and non-severe stage 2 *g*-HAT, for whom both fexinidazole and pentamidine/NECT are indicated [1, 3]. We considered patients who did not undergo LP (i.e. patients without neurological signs or symptoms compatible with severe stage 2) as affected by stage 1 or non-severe stage 2 *g*-HAT. Adverse drug reactions were defined as signs or symptoms not present before treatment beginning and likely due to drug administration. Concomitant treatments were defined as drugs administered for causes other than ADRs and malaria.

### Study setting

Health service in South Sudan is delivered through the following decentralized structures: Primary Health Care Units (PHCUs), Primary Health Care Centers (PHCCs), County and State Hospitals: PHCUs are located in Bomas (i.e. villages), PHCCs are usually located in Payams (i.e. the first level of county subdivision), and County Hospitals are located in County administrative headquarters. County is the main subdivision of a State.

The study involved *g*-HAT patients admitted to Lui Hospital, Mundri East County, Western Equatoria State, South Sudan. Lui Hospital is the referral hospital for Mundri East, Mundri West and Mvolo, three Counties of Western Equatoria State, serving a population of almost 208,426 people. Additionally, individuals from further Counties also seek care at Lui Hospital. It is a public-private hospital belonging to the South Sudanese Episcopal Church and recognized by the government as a key component of the health system in this region. The hospital has 120 beds and is divided into medical, surgical, maternity, and pediatric wards. Doctors with Africa CUAMM, an Italian NGO, started supporting Lui Hospital in 2009, taking over its management from mid-2016 to mid-2024.

At Lui Hospital, *g*-HAT diagnostic pathway was led according to 2019 South Sudan Standard Treatment and 2019 WHO Guidelines [1, 24]. Depending on laboratory availability, either the RDT (SD Bioline HAT, Abbott Diagnostics, South Korea) or the CATT (CATT/*T. b. gambiense*, Institute of Tropical Medicine, Antwerp) is initially requested for suspected *g*-HAT patients to rule out the infection. In case of positivity, confirmation of the diagnosis relies on Woo test, performed on capillary or venipuncture peripheral blood. Approximately 50 𝜇L of blood are collected in capillary tubes containing an anticoagulant; after sealing, the sample is centrifugated at high-speed (12,000 g for 5 min) in a haematocrit centrifuge, and then examined at low magnification (10 × 10) for mobile parasites at the junction between WBC and plasma layers [13]. In case of palpable neck lymph nodes, they are punctured to obtain fresh lymph, which is microscopically examined directly at 10 × 40 magnification. In case of positivity of Woo test or gland puncture examination, LP is performed: however, after the introduction of fexinidazole, LP is performed only in case of neurological signs or symptoms compatible with severe stage 2 *g*-HAT.

Once the disease stage is defined, the patient is treated according to the 2019 South Sudan Standard Treatment and 2019 WHO Guidelines [1, 24], based on drug availability. Fexinidazole tablets have been delivered to the hospital since 20/12/2022 by WHO and Ministry of Health with rare shortage of supplies. Pentamidine, NECT, and melarsoprol were regularly supplied to the hospital before and after fexinidazole introduction.

### Data collection

We analyzed patient data by consulting patient medical records, Lui Hospital ward registers (including outpatient department, medical, surgical, pediatric, and maternity wards), pharmacy records, and HAT surveillance registers concerning demographic, clinical, diagnostic, therapeutic data, and outcomes. For 26 patients, only demographic and laboratory data were available from the HAT surveillance register, as clinical records were missing in the hospital archives. The anonymized data were subsequently entered into a computerized database (.xlsx file, Microsoft Excel 365^®^).

All data previously collected in this research project were processed according to current privacy regulations and in line with the recommendations governing good clinical practice. Data were collected in the Excel spreadsheet anonymously, and each patient was assigned a progressive number.

### Data analysis

**Fig. 2:**
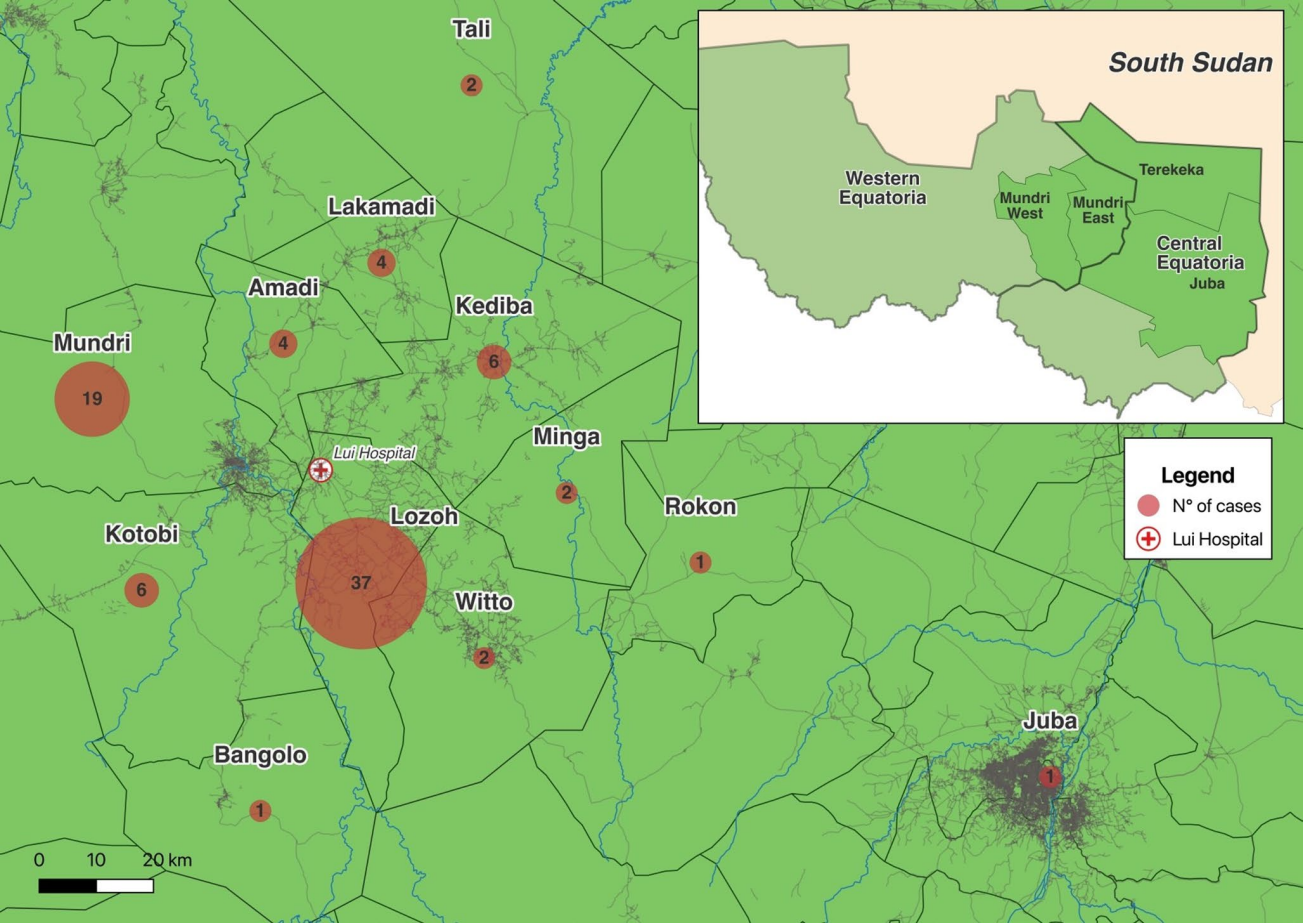
Distribution of *gambiense* human African trypanosomiasis confirmed cases in Lui Hospital, Western Equatoria, South Sudan (July 2018-June 2024), divided by Payams and Counties of provenience

Descriptive analysis was employed to illustrate population characteristics in overall population and in patients eligible for fexinidazole. Statistical analysis was conducted only on the 46 patients eligible for fexinidazole. Categorical variables were evaluated using *X*2 test; Fisher’s exact test was used when at least one element of contingency tables was < 5. Continuous variables were analyzed using the Mann-Whitney test. Considering the small size of the population, logistic regression with the Firth model was used in patients eligible for analysis to assess significant correlation with unfavourable outcome and both discrete and continuous variables (age, disease stage, abnormal neurological examination at admission, and administration of fexinidazole). STATA v18.0 (STATACorp, College Station, TX, USA) was used for statistical analyses. A *p*-value less than 0.05 was considered statistically significant. QGIS v3.34.8-Prizren was used to realize Fig. 2.

### Ethical considerations

The study was conducted in accordance with the Declaration of Helsinki and the 2019 National Guidelines for Research Involving Humans in the Republic of South Sudan [25]. Being a study based on existing and routinely available data, patients were did not directly involved and no informed consent was required, in accordance with the 2019 National Guidelines for Research Involving Humans in the Republic of South Sudan [25] and South Sudan Ministry of Health. The transmission or dissemination of data was approved by the South Sudan Ministry of Health Ethical Committee (MOH/RERB/D.3/2024, 15th April 2024).

## Results

During the study period, 1,553 *g*-HAT serological tests (RDT or CATT) were performed as passive screening in patients admitted to Lui Hospital: 94 patients resulted positive to serological tests, while 86 (86/1,553, 5.5%) were confirmed as *g*-HAT cases (Supplementary Figure). Among them, 44/86 patients (51.2%) were male, and the median age was 26 years [IQR 19.0–40.0]. Payams of origin are described in Fig. 2: Counties of origin were Mundri East (52), Mundri West (30), Juba (2), Terekeka (2).

Eleven out of 86 (12.8%), 41/86 (47.7%), and 21/86 (24.4%) patients were diagnosed with stage 1, non-severe stage 2, and severe stage 2 *g*-HAT, respectively. Lumbar puncture was not performed in 13 (15.1%) patients: 4 of them refused admission to the hospital, and staging was not available, while the remaining 9 patients did not undergo LP (patients without LP - pwLP) due to the absence of neurological signs or symptoms compatible with severe stage 2 *g*-HAT.

Among the general population, complete clinical and therapeutic documentation was available for 60 patients, and 46 of them were eligible for fexinidazole (Fig. 1). Main demographic, clinical, and therapeutic differences are shown in Supplementary Table.

Of the 46 patients, 9 were diagnosed with stage 1 *g*-HAT (19.6%), 30 with non-severe stage 2 *g*-HAT (65.2), and 7 were assigned to pwLP (15.2%). Patients treated with fexinidazole and pentamidine/NECT did not significantly differ in terms of sex, age, CSF findings, presence of CNS symptoms at admission, and malaria co-infection. After fexinidazole introduction in Lui Hospital, LP was performed in 29/36 (80.6%) patients eligible for fexinidazole (i.e. absence of symptoms/signs compatible with severe stage 2 *g*-HAT); in contrast, before fexinidazole availability, 10/10 (100.0%) patients underwent LP since pentamidine/NECT were the only treatments available. Presence of abnormal neurological examination at admission was significantly more frequent in patients treated with fexinidazole (6.7% vs. 0.0%, *p* = 0.022). Median duration of hospitalization was 10 days [IQR 10–12]. At discharge, we observed persistence of symptoms in 20.0% and 6.3% of patients treated with fexinidazole and pentamidine/NECT, respectively. Abnormal neurological examination was evidenced in 6.7% and 6.3% of patients treated with fexinidazole and with pentamidine/NECT, respectively: in the second cohort, abnormal neurological signs occurred during hospitalization. In-hospital deaths were not described (Table 1).

**Table 1:** Demographic, clinical, therapeutic variables, and outcomes of patients with stage 1 and non-severe stage 2 *gambiense* human African trypanosomiasis diagnosed in Lui hospital, Western equatoria, South Sudan (July 2018-June 2024), with available clinical charts, divided by treatment (fexinidazole vs. treatments other than fexinidazole)

	Fexinidazole (*n* = 30)	Other treatments^1^ (*n* = 16)	*p*-value
Male, *n* (%)	12 (40.00)	8 (50.00)	0.515
Age, median years [IQR]	23.0 [19.0–39.0]	29.5 [18.0-42.5]	0.791
CSF findings			
*WBC in CSF*, *median cells/µL [IQR]*	53.0 [12.0–89.0]	40.0 [12.0–72.0]	0.474
*Trypanosoma in CSF*, *n (%)*	0 (0.00)	1 (6.25)	0.410
Disease stage, *n* (%)			
*1*	5 (16.67)	4 (25.00)	0.139
*2 non-severe*	18 (60.00)	12 (75.00)	
*Not performed LP*^*2*^	7 (23.33)	0 (0.00)	
CNS symptoms at admission, *n* (%)	29 (96.67)	14 (93.33)	1.000
Abnormal neurological examination at admission, *n* (%)	2 (6.67)	0 (0.00)	**0.022**
Malaria co-infection, *n* (%)	7 (23.33)	3 (18.75)	1.000
Unfavourable outcome at discharge, *n* (%)	7 (23.33)	1 (6.25)	0.230
*Symptoms persistence*, *n (%)*	6 (20.00)	1 (6.25)	0.282
*Headache*, *n (%)*	4 (13.33)	0 (0.00)	0.282
*Behaviour disorders*, *n (%)*	2 (6.67)	0 (0.00)	0.536
*Speech disorders*, *n (%)*	1 (3.33)	1 (6.25)	1.000
*Abnormal neurological examination*, *n (%)*	2 (6.67)	1 (6.25)	0.592
ADRs, *n* (%)	21 (70.00)	8 (50.00)	0.181
*Gastrointestinal*, *n (%)*	19 (63.33)	3 (18.75)	**0.005**
*Asthenia*, *n (%)*	0 (0.00)	1 (6.25)	0.348
*Headache*, *n (%)*	2 (6.67)	1 (6.25)	1.000
*Hypertension*, *n (%)*	2 (6.67)	2 (12.50)	0.602
*Pruritus*, *n (%)*	2 (6.67)	0 (0.00)	0.536
*CNS*, *n (%)*	3 (10.00)	3 (18.75)	0.405
*Psychiatric*, *n (%)*	1 (3.33)	0 (0.00)	1.000
Treatment discontinuation, *n* (%)	0 (0.00)	1 (6.25)	0.348

Bold *p*-values are statistically significant

^1^ Other treatments are comprehensive of Nifurtimox Eflornithine Combination Therapy (NECT), NECT long and pentamidine;

^2^ LP was not performed since patients did not show neurological signs and symptoms compatible with severe stage 2 *g*-HAT

ADRs: adverse drug reactions; CNS: central nervous system; CSF: cerebrospinal fluid; IQR: interquartile range; WBC: white blood cells

An unfavourable outcome was present in 8/46 patients (7 treated with fexinidazole and 1 treated with pentamidine/NECT). It was significantly more frequent in pwLP (50.0% vs. 7.9%, *p* = 0.026) (Table 2): among them, 4/7 experienced an unfavourable outcome, due to persistence of headache (75.0%) and to occurrence of sleeping disorders (25.0%) during hospitalisation. Logistic regression did not show any correlation between unfavourable outcome and age, use of fexinidazole, abnormal neurological examination at admission, and stage of disease (pwLP were not included) (Table 3).

**Table 2:** Demographic, clinical, and therapeutic characteristics of patients with *gambiense* human African trypanosomiasis diagnosed in Lui hospital, Western equatoria, South sudan, from July 2018 to June 2024 with available clinical charts and eligible for fexinidazole, divided by favourable and unfavourable outcomes

Patients eligible for fexinidazole	Favourable outcome^1^	Unfavourable outcome^2^	*p*-value
	*n* = 38	*n* = 8	
Male, *n* (%)	16 (42.11)	4 (50.00)	0.713
Age, median years [IQR]	23.0 [19.0–40.0]	34.5 [21.0–54.0]	0.234
Kind of referral, *n* (%)			
*Direct access*	23 (60.53)	7 (87.50)	0.352
*PHCUs*	4 (10.53)	1 (12.50)	
*PHCCs*	10 (26.32)	0 (0.00)	
*Other Hospitals*	1 (2.63)	0 (0.00)	
CSF findings			
*Performed LP*, *n (%)*	35 (92.11)	4 (50.00)	**0.012**
*WBC in CSF*, *median cells/µL [IQR]*	42.0 [12.0–72.0]	89.5 [46.5–96.5]	0.131
*Trypanosoma in CSF*, *n (%)*	1 (2.86)	0 (0.00)	1.000
Disease stage, *n* (%)			
*1*	8 (21.05)	1 (12.50)	**0.026**
*2 non-severe*	27 (71.05)	3 (37.50)	
*Not performed LP*^*3*^	3 (7.89)	4 (50.00)	
CNS symptoms at admission, *n* (%)	36 (97.30)	7 (87.50)	0.327
*Sleeping disorders*, *n (%)*	13 (35.14)	1 (12.50)	0.402
*Headache*, *n (%)*	25 (67.57)	6 (75.00)	1.000
*Behaviour disorders*^*4*^, *n (%)*	15 (39.47)	2 (25.00)	0.691
*Motor disorders*^*5*^, *n (%)*	9 (23.68)	3 (37.50)	0.412
*Seizures*, *n (%)*	3 (8.11)	1 (12.50)	0.557
*Confusion or unconsciousness*, *n (%)*	17 (44.74)	4 (50.00)	1.000
*Disorders of memory*, *n (%)*	3 (8.11)	0 (0.00)	1.000
Abnormal neurological examination at admission, *n* (%)	1 (2.63)	1 (12.50)	0.237
Fexinidazole administered, *n* (%)	23 (60.53)	7 (87.50)	0.230
Latency between symptoms and treatment^7^, median days [IQR]	25.5 [7.5-190.5]	10.0 [6.0-240.0]	0.934
Malaria co-infection, *n* (%)	9 (23.68)	1 (12.50)	0.664
Concomitant treatments, *n* (%)	17 (44.74)	4 (50.00)	1.000

Bold *p*-values are statistically significant. ^1^ Favourable outcome is defined as absence of signs and symptoms consistent with *gambiense* human African trypanosomiasis (*g*-HAT) at the discharge;

^2^ Unfavourable outcome is defined as presence of signs or symptoms consistent with *g*-HAT at the discharge, or death related to *g*-HAT or *g*-HAT treatment occurred during hospitalisation;

^3^ LP was not performed since patients did not show neurological signs and symptoms compatible with severe stage 2 *g*-HAT;

^4^ Behavior disorders were intended as described different personality feature, aggressiveness, restlessness;

^5^ Motor disorders were intended as movement, speech disorders and asthenia;

^6^ Data about latency were available for 35 patients (28 with favourable outcome, 7 with unfavourable outcome)

CNS: central nervous system; CSF: cerebrospinal fluid; IQR: interquartile range; LP: lumbar puncture; NECT: nifurtimox eflornithine combination therapy; PHCCs: primary health care centers; PHCUs: primary health care units; WBC: white blood cells

**Table 3:** Logistic regression with the Firth model used to examine the association between unfavourable outcome and selected variables (age, stage, abnormal neurological examination at admission, and use of fexinidazole) in patients diagnosed with stage 1 and non-severe stage 2 *gambiense* human African trypanosomiasis admitted to Lui hospital, Western equatoria, South Sudan (July 2018-June 2024)

	OR	*p*-value	[95% CI]
Age	1.070	*p* = 0.051	[1.000–1.146]
Non-severe stage 2^1^	0.991	*p* = 0.995	[0.075–13.123]
Fexinidazole treatment	1.213	*p* = 0.877	[0.104–14.122]
Admission abnormal neurological examination	26.296	*p* = 0.061	[0.860–803.669]

^1^ Patients in which lumbar puncture was not performed were excluded from the analysis. The analysis was hence run over 39 patients

Twenty-nine patients (29/46, 63.0%) presented ADRs: the most frequent ADRs were gastro-intestinal (GI) (47.8%), CNS (13.0%), hypertension (8.7%), headache (6.5%), and pruritus (4.4%). Patients experiencing ADRs did not differ from patients without ADRs in terms of sex, age, stage of disease, malaria co-infection, concomitant treatments, and use of fexinidazole (Table 4). No statistically significant differences in ADRs frequency were evidenced between fexinidazole and pentamidine/NECT regimens (Table 1). Patients treated with fexinidazole experienced GI ADRs (i.e. nausea, vomiting and abdominal pain) in 63.3% of cases, significantly more than those treated with pentamidine/NECT (18.8%, *p* = 0.005). Non-GI ADRs were mainly present in patients treated with pentamidine/NECT regimens, without a statistically significant difference (30.0% vs. 37.5%, *p* = 0.605). One patient in treatment with NECT discontinued treatment (Table 1).

**Table 4:** Demographic, clinical, therapeutic characteristics, and outcome of patients diagnosed with *gambiense* human African trypanosomiasis in Lui hospital, Western equatoria, South Sudan (July 2018-June 2024), eligible for fexinidazole, divided by presence or absence of ADRs

	Presence of ADRs (*n* = 29)	Absence of ADRs (*n* = 17)	*p*-value
Male, *n* (%)	12 (41.38)	8 (47.06)	0.708
Age, median years [IQR]	23.0 [19.0–43.0]	25.0 [19.0–34.0]	0.600
Disease stage, *n* (%)			
*1*	5 (17.24)	4 (23.53)	0.738
*2 non-severe*	20 (68.97)	10 (58.82)	
*Not performed LP*^*1*^	4 (13.79)	3 (17.65)	
Fexinidazole administered, *n* (%)	21 (72.41)	9 (52.94)	0.181
Pentamidine/NECT administered, *n* (%)	8 (27.59)	8 (47.06)	
Malaria co-infection, *n* (%)	7 (24.14)	3 (17.65)	0.723
Concomitant treatments, *n* (%)	14 (48.28)	7 (41.18)	0.641
Unfavourable outcome^2^, *n* (%)	7 (24.14)	1 (5.88)	0.226

^1^ LP was not performed since patients did not show neurological signs and symptoms compatible with severe stage 2 *g*-HAT

^2^ Unfavourable outcome is defined as presence of signs or symptoms consistent with *g*-HAT at the discharge or death related to *g*-HAT or *g*-HAT treatment occurred during hospitalisation

ADRs: adverse drug reactions; LP: lumbar puncture; IQR: interquartile range; NECT: Nifurtimox Eflornithine Combination Therapy

## Discussion

In South Sudan, *g*-HAT foci are mainly distributed in the Equatoria States bordering Uganda, Democratic Republic of Congo and Central African Republic, in a geographical belt from Kajo Keji County (Eastern Equatoria State) to Tambura County (Western Equatoria State): the incidence of the disease experienced three main peaks in the past 70 years, determined by mass population migrations and the withdrawal of NGOs from screening programs [2, 26]. According to WHO, less than 50 new cases per year were reported in South Sudan from 2018: 17 (2018), 11 (2019), 15 (2020), 10 (2021), 30 (2022), and 50 (2023) [27]. Lui Hospital collects patients mainly from 3 Counties (Mundri East, Mundri West and Mvolo): Greater Mundri area is historically known as *g*-HAT focus, with a prevalence of 0.46% in 2008 [12, 23]. The number of new patients diagnosed with *g*-HAT in Lui Hospital was in line with the trend of national cases: 4 (2018), 0 (2019), 7 (2020), 2 (2021), 20 (2022), and 43 (2023); until June 2024, 10 new cases of *g*-HAT have been diagnosed.

The majority of the study population presented directly to the hospital (66.7%), reflecting a scarcity of active screening programs and an approach substantially based on passive case finding. The population was mostly represented by young patients with non-severe stage 2 *g*-HAT, with a median latency from symptoms onset to treatment beginning of almost 25 days.

The discharge outcomes of patients treated with fexinidazole and pentamidine/NECT were compared, analysing patients with stage 1, non-severe stage 2 *g*-HAT, and without signs or symptoms compatible with severe stage 2, for which both fexinidazole and pentamidine/NECT treatments are currently indicated. Results concerning outcome at discharge showed a trend of persistence of symptoms or signs compatible with *g*-HAT slightly higher in fexinidazole cohort than in pentamidine/NECT cohort (20.0% vs. 6.2%): this observation did not reach statistical significance (*p* = 0.230) and still study population is too small to draw consistent conclusions. Particularly, headache was the main persistent symptom (13.3% in fexinidazole cohort). Persistence of symptoms (especially headache and pruritus) has been reported at the end of treatment and after 3 months in *g*-HAT patients treated with pentamidine (stage 1) and melarsoprol (stage 2) [28]; to our knowledge, no similar data for NECT or fexinidazole are available. Fexinidazole cohort showed a higher percentage of neurological abnormalities at admission compared to the pentamidine/NECT cohort (6.7% vs. 0.0%, *p* = 0.022), a result that possibly influenced the final results. Our assessment was done at the discharge of the patient and is not directly comparable to the 18-month outcomes exposed in literature [15, 16, 32].

According to the 2019 WHO guidelines, since the introduction of fexinidazole at Lui Hospital in December 2022, we have performed LP only in patient with CNS signs or symptoms compatible with stage 2 severe *g*-HAT [1]. In 15.2% of the patients eligible for fexinidazole LP was not performed (pwLP): of them, 4/7 (57.1%) had an unfavourable outcome, mainly due to the persistence of headache (75%). This observation could underline the importance of accurately selecting clinical features that necessitate LP, despite more studies are needed.

According to logistic regression, age, stage of disease, fexinidazole administration and abnormal neurological examination at admission did not have significant impact on the outcome. However, results could be influenced by the small size of the population.

Among patients eligible for fexinidazole, 63% experienced at least one ADR. Patients treated with fexinidazole experienced more ADRs compared to patients treated with pentamidine/NECT (70.0% vs. 50.0%, *p* = 0.181), although this was not statistically significant. Mesu et al. reported a higher rate of ADRs in patients treated with fexinidazole (90–94%): this difference may be partially explained by the unavailability at Lui Hospital of blood test for electrolytes, albuminemia, neutrophil count, and other parameters possibly influenced by fexinidazole [15, 16, 17]. In the fexinidazole cohort, the most common ADRs were GI (63.3%), significantly higher than in the pentamidine/NECT group (18.8%, *p* = 0.005), in line with the frequency of GI ADRs in fexinidazole-treated patients described in the literature (60–78%) [15, 16]. Non-GI ADRs evidenced in fexinidazole group were CNS (10.0%), hypertension (6.7%), headache (6.7%), and pruritus (6.7%), in line with the literature. We did not observe any discontinuation of the drug in the fexinidazole cohort, in contrast with the pentamidine/NECT cohort, where 1 patient discontinued treatment.

To our knowledge, this is the first real-life observation of fexinidazole use for *g*-HAT in South Sudan. Fexinidazole showed: (1) optimal discharge outcome, lower but not significantly different from those of the pentamidine/NECT group; (2) a relatively high rate of GI ADRs without any treatment discontinuation. Moreover, after fexinidazole introduction in Lui Hospital, LP was performed in 80.6% patients eligible for fexinidazole (i.e. absence of symptoms/signs compatible with severe stage 2 *g*-HAT), in contrast with 100.0% of patients before fexinidazole availability. The reduction in the frequency of LP highlights how fexinidazole could simplified *g*-HAT management, particularly in low-resources settings. According to Das et al., the introduction of a completely oral therapy could improve patient compliance, considering the possibility of outpatient management of the disease: in this setting, directly observed therapy (DOT) could be a useful resource to monitor the correct administration of the treatment [1, 3, 29]. WHO guidelines suggest DOT for patients trusted to be compliant, to have food, and without psychiatric disorders [1, 3]; in our rural setting, we decided to administer fexinidazole entirely during the hospitalisation due to the remoteness of patient villages and the poor organization of peripheral health-care facilities. We strongly believe that the use of DOT could be an important advantage in a well-structured peripheral health-care system: its use in remote villages where hospitalisation is not feasible could increase the number of treated patients, mainly in stage 1 (i.e. the stage in which the trypanosome is more susceptible to being transmitted), leading to a decline in the epidemiological curve [29, 30]. This approach should be combined with active case-finding through screening campaigns to identify symptomatic and oligosymptomatic patients who are thought to mainly contribute to the transmission of the disease [31].

Our study has several limitations. Firstly, it is a monocentric retrospective cohort study, and complete clinical and therapeutic data were not available for 26/86 patients. The population eligible for analysis was composed by only 46 patients: despite the use of statistical tests suitable for small populations, the results cannot lead to any consistent conclusion. The rural context and the absence of funding did not allow the follow-up of our patients for at least 24 months as advised by WHO [1, 3], and our outcome (clinical assessment at discharge) is not directly comparable to the 18-months registered efficacy described in the literature [15, 16, 32]. Moreover, the unavailability of several blood tests may have led to a potential underestimation of ADRs.

## Conclusion

Fexinidazole showed a favourable outcome at discharge comparable to pentamidine/NECT. Despite a higher frequency of ADRs, mostly GI, no patient discontinued the treatment prematurely. The lack of treatment discontinuation and the reduced need for LP make fexinidazole an excellent drug, allowing DOT in carefully selected patients in rural settings.

## Supplementary Information

Below is the link to the electronic supplementary material.

Supplementary figure: Trends of passive screening tests and confirmed *gambiense* human African trypanosomiasis cases performed in Lui Hospital, Western Equatoria, South Sudan (July 2018-June 2024). Footnotes: *g*-HAT: *gambiense* human African trypanosomiasis

Supplementary Table: Demographic, clinical, therapeutic characteristics, ADRs, and outcome at discharge of patients with gambiense human African trypanosomiasis diagnosed in Lui Hospital, Western Equatoria, South Sudan, from July 2018 to June 2024 (A), with available clinical charts and eligible for fexinidazole (B)

Supplementary Material: STROBE Statement—Checklist of items that should be included in reports of cohort studies

## Data Availability

No datasets were generated or analysed during the current study.
