# Supplementary figures and images for: Use of fexinidazole in gambiense human African trypanosomiasis: a retrospective analysis of cases treated in Lui Hospital, South Sudan (2018–2024)
Source: Infection. 2025 Sep 4;53(6):2847–57. doi: 10.1007/s15010-025-02633-6 (PMC12675666; doi:10.1007/s15010-025-02633-6)

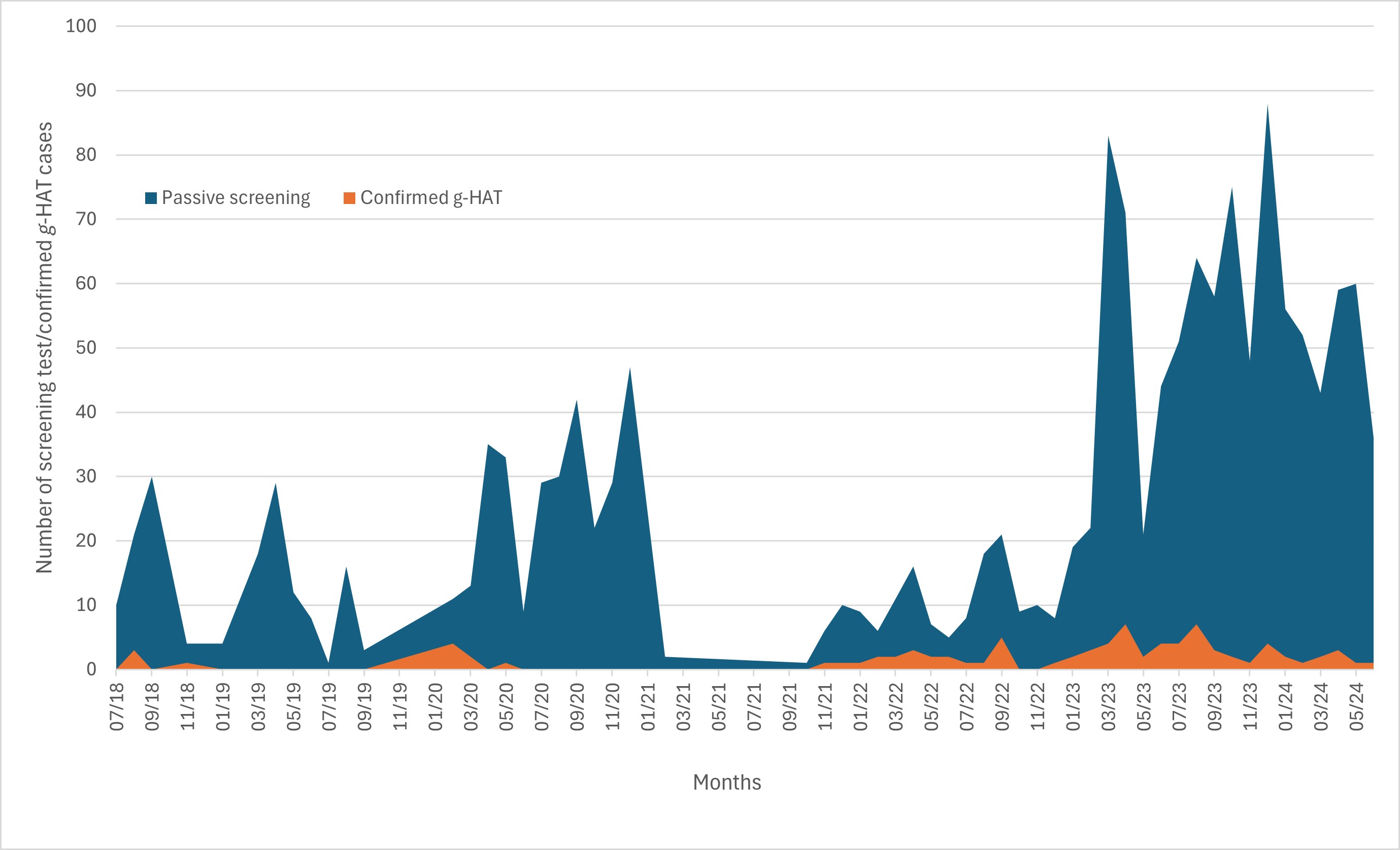

Supplement: Supplementary file 1 — Supplementary figure: Trends of passive screening tests and confirmed gambiense human African trypanosomiasis cases performed in Lui Hospital, Western Equatoria, South Sudan (July 2018-June 2024). Footnotes: g-HAT: gambiense human African trypanosomiasis [file 15010_2025_2633_MOESM1_ESM.jpeg]
