# Supplementary material for: Use of fexinidazole in gambiense human African trypanosomiasis: a retrospective analysis of cases treated in Lui Hospital, South Sudan (2018–2024)
Source: Infection. 2025 Sep 4;53(6):2847–57. doi: 10.1007/s15010-025-02633-6 (PMC12675666; doi:10.1007/s15010-025-02633-6)
Supplement: Supplementary file 2 — Supplementary Table: Demographic, clinical, therapeutic characteristics, ADRs, and outcome at discharge of patients with gambiense human African trypanosomiasis diagnosed in Lui Hospital, Western Equatoria, South Sudan, from July 2018 to June 2024 (A), with available clinical charts and eligible for fexinidazole (B) [file 15010_2025_2633_MOESM2_ESM.docx]

**Supplementary Table.** Demographic, clinical, therapeutic characteristics, ADRs, and outcome at discharge of patients with *gambiense* human African trypanosomiasis diagnosed in Lui Hospital, Western Equatoria, South Sudan, from July 2018 to June 2024 (A), with available clinical charts and eligible for fexinidazole (B).

|  | A  (n= 86) | B  (n= 46) |
| --- | --- | --- |
| Male, n (%) | 44 (51.16) | 20 (43.48) |
| Age, median years [IQR] | 26.0 [19.0-40.0] | 24.0 [19.0-40.0] |
| Kind of referral, n (%) | | |
| *Direct access* | 43/64 (67.19) | 30 (65.22) |
| *PHCUs* | 6/64 (9.38) | 5 (10.87) |
| *PHCCs* | 14/64 (21.88) | 10 (21.74) |
| *Other Hospitals* | 1/64 (1.56) | 1 (2.17) |
| CSF findings | | |
| *Performed LP, n (%)* | 73 (84.88) | 39 (84.78) |
| *WBC in CSF, median cells/µL [IQR]* | 66.0 [20.0-103.0] | 43.0 [12.0-82.0] |
| *Trypanosoma in CSF, n (%)* | 13/72 (18.06) | 1/39 (2.56) |
| Disease stage, n (%) | | |
| *1* | 11 (12.79) | 9 (19.57) |
| *2 non-severe* | 41 (47.67) | 30 (65.22) |
| *2 severe* | 21 (24.42) | - |
| *Not performed LP* | 13 (15.12) | 7 (15.22) |
| CNS symptoms at admission, n (%) | 60/62 (96.77) | 43/45 (95.56) |
| Abnormal neurological examination at admission, n (%) | 4/63 (6.35) | 2 (4.35) |
| Treatment administered, n (%) | | |
| *Fexinidazole, n (%)* | 32/64 (50.00) | 30 (65.22) |
| *Pentamidine, n (%)* | 3/64 (4.69) | 3 (6.52) |
| *NECT, n (%)* | 29/64 (45.31) | 13 (28.26) |
| Malaria co-infection, n (%) | 19/61 (31.15) | 10 (21.76) |
| Concomitant treatments, n (%) | 31/60 (51.67) | 21 (45.65) |
| Unfavourable outcome^1^ at discharge, n (%) | 13/63 (20.63) | 8 (17.39) |
| ADRs, n (%) | 38/60 (63.33) | 29 (63.04) |
| Treatment discontinuation, n (%) | 3/59 (5.08) | 1 (2.17) |

^1^ Unfavourable outcome is defined as presence of signs or symptoms consistent with *g*-HAT at discharge, or death related to *g*-HAT or *g*-HAT treatment occurred during hospitalisation.
ADR: adverse drug reactions; CNS: central nervous system; CSF: cerebrospinal fluid; IQR: interquartile range; LP: lumbar puncture; NECT: nifurtimox eflornithine combination therapy; PHCCs: primary health care centers; PHCUs: primary health care units; WBC: white blood cells.
